# Deletion of *NTH1* and *HSP12* increases the freeze–thaw resistance of baker’s yeast in bread dough

**DOI:** 10.1186/s12934-022-01876-4

**Published:** 2022-07-25

**Authors:** Bo-Chou Chen, Huan-Yu Lin

**Affiliations:** Bioresource Collection and Research Center, Food Industry Research and Development Institute, Hsinchu, 300 Taiwan

**Keywords:** *S. cerevisiae*, *NTH1*, *HSP12*, Freeze–thaw resistance, CRISPR, Cpf1

## Abstract

**Background:**

The intracellular molecule trehalose in *Saccharomyces cerevisiae* may have a major protective function under extreme environmental conditions. *NTH1* is one gene which expresses trehalase to degrade trehalose. Small heat shock protein 12 (*HSP12* expressed) plays a role in protecting membranes and enhancing freezing stress tolerance.

**Results:**

An optimized *S. cerevisiae* CRISPR-Cpf1 genome-editing system was constructed. Multiplex genome editing using a single crRNA array was shown to be functional. *NTH1* or/and *HSP12* knockout in *S. cerevisiae* enhanced the freezing stress tolerance and improved the leavening ability after freezing and thawing.

**Conclusions:**

Deleting *NTH1* in the combination with deleting *HSP12* would strengthen the freezing tolerance and protect the cell viability from high rates of death in longer-term freezing. It provides valuable insights for breeding novel *S. cerevisiae* strains for the baking industry through a more precise, speedy, and economic genome-editing system.

## Background

Frozen dough technology is important to the development of the bakery industry because the frozen dough can be delivered to downstream sales at low temperatures, and through simple thawing, fermentation, and baking, it is convenient to supply freshly baked bread to consumers. The technology not only can provide consumers with freshly baked products at any time, but also reduce economic losses caused by unstable quality, short shelf life, and aging of baked products. However, a disadvantage of the frozen dough technology is the low resistance of baker’s yeast, *Saccharomyces cerevisiae*, to freezing stress [1]. Considerable evidence indicates that the intracellular molecule trehalose in *S. cerevisiae* may have a major protective function under extreme environmental conditions [2]. Trehalose in *S. cerevisiae* can maintain the membrane integrity by combining with phospholipids and the native conformation of proteins, preventing the aggregation of partially denatured proteins to achieve the effect of freezing resistance [3, 4].

The biosynthesis of trehalose is a two-step process, involving the production of trehalose-6-phosphate (T6p) catalyzed by trehalose-6-phosphate synthase (Tps) and its consecutive dephosphorylation to trehalose, catalyzed by trehalose-6-phosphate phosphatase (Tpp) [5–7]. However, the intracellular level of trehalose in yeast cells is the result of a well-regulated balance between enzymatic synthesis and degradation [8]. Trehalose is hydrolyzed into glucose by a cytosolic/neutral trehalase encoded by *NTH1* [9, 10]. Deletion of *NTH1* results in decreased trehalase activity, enhanced accumulation of trehalose, and increased freezing resistance [11–13]. Except for *NTH1*, there are two genes, *NTH2* and *ATH1*, also involved in the hydrolysis of trehalose. The *NTH2* gene, a paralog of *NTH1*, encodes a functional trehalase involved in trehalose mobilization [14]. An acid trehalase encoded by *ATH1*, whose major activity is measured extracellularly because of the vacuolar localization. Lack of Ath1p does not alter intracellular levels of trehalose [15, 16].

Accumulated evidence indicates that molecular chaperones such as the heat shock family of stress proteins (HSPs) actively participate in an array of cellular processes, including cryoprotection [17]. Small heat shock protein 12 (Hsp12p) plays a role in protecting membranes against desiccation and ethanol-induced stress [18], enhancing osmotolerance [19], and freezing tolerance [20]. The *HSP12* deletion mutant is more resistant to freezing, and overexpression of Hsp12p in the *TPS1* deletion strain increases in resistance to freezing storage and heat stress [20].

The clustered regularly interspaced short palindromic repeats (CRISPR) as a bacterial immune defense system that has been developed for genome-editing tools in a variety of organisms [21]. The class 2 CRISPR-Cas9 system is widely used for genome editing due to the inherent simplicity and flexibility in sequence requirements for single guide RNA (sgRNA). Using Cas9 endonuclease from *Streptococcus pyogenes*, a sgRNA containing 20 bp complementary sequence of the target sequence can guide the Cas9 to target DNA with a protospacer adjacent motif (PAM) to generate a double-strand break [21]. The microorganisms with broken DNA prefer to repair by homologous recombination with homologous DNA templates [21, 22]. Although the CRISPR-Cas9 system has been adapted for genome editing of multiple microorganisms, its use is restricted because of the toxicity of Cas9 and a G-rich PAM sequence. CRISPR-Cpf1 is a novel, simple, and efficient genome-editing tool for *S. cerevisiae* [23]. Cpf1 is an RNA-guided endonuclease of class 2 CRISPR-Cas systems that cleaves target DNA with features different from those of Cas9 [24, 25], such as utilizing a T-rich PAM [26, 27], guided by one single crRNA [28, 29], and DNA cleavage results in sticky ends [27].

In the present study, we first generated a CRISPR-Cpf1 genome-editing system in *S. cerevisiae* using a visual recognition model that presents red colonies when the *ADE2* gene is deleted [30]. Furthermore, we constructed baker’s yeast strains with deleted trehalase gene and/or heat shock protein 12 gene by CRISPR-Cpf1 system to investigate the content of intracellular trehalose, and cell viability after freezing and thawing. Finally, the leavening ability of the frozen bread dough with edited yeast strains was analyzed.

## Results

### Optimization of the CRISPR-Cpf1 genome-editing system in *S. cerevisiae*

**Fig. 1:**
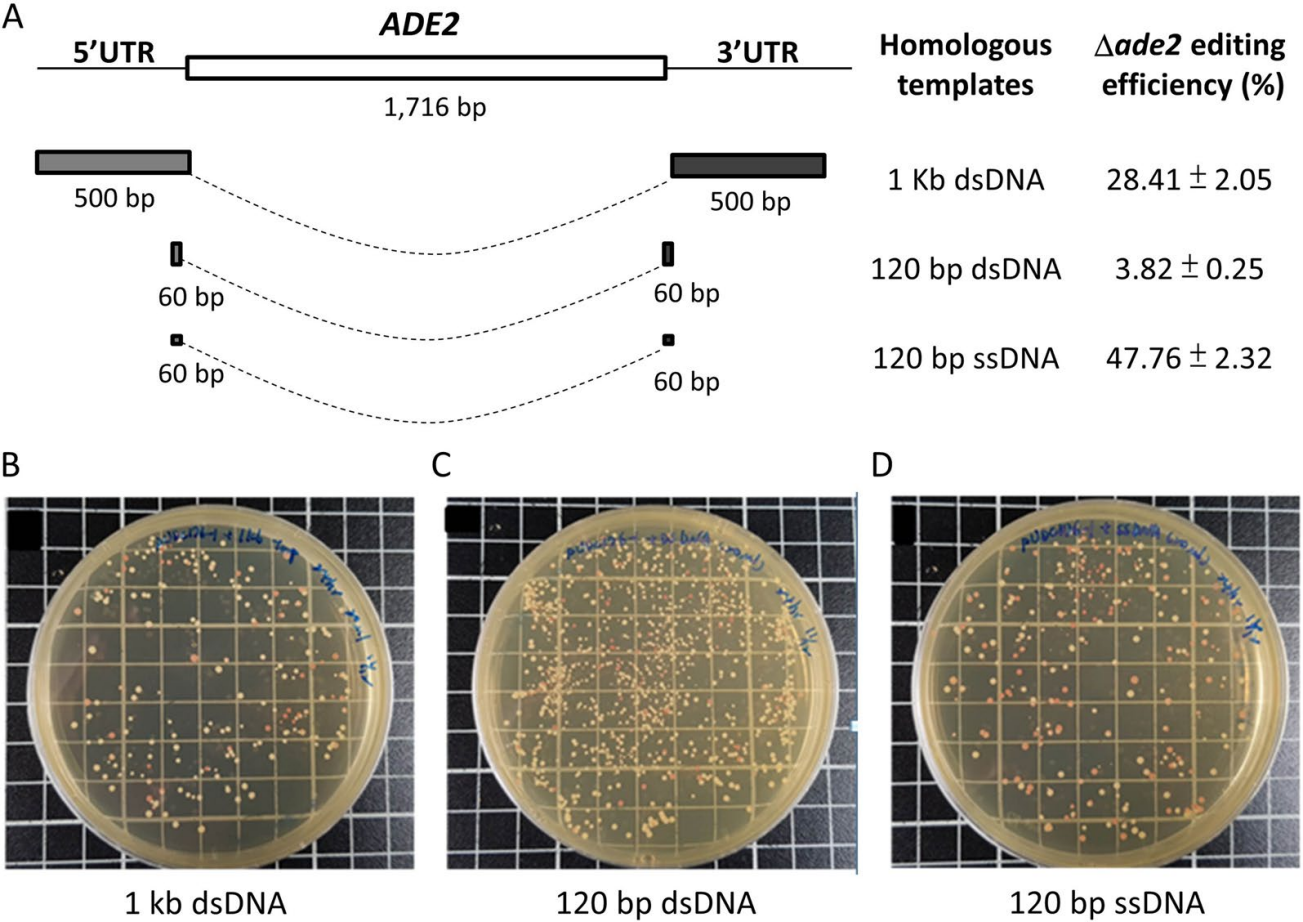
The homologous recombination DNA templates for *ADE2* in CRISPR-Cpf1 system. **A** Scheme showing the design of the homologous recombination DNA templates for *ADE2* gene and the genome-editing efficiency using CRISPR-Cpf1 in *S. cerevisiae* system. Data were derived from three separate experiments and are presented as the means ± SD. **B**–**D** Colonies of the strains with *ADE2* gene disruption were shown in red color. The homologous recombination DNA templates used for CRISPR editing were **B** 1 kb dsDNA, **C** 120 bp dsDNA, and **D** 120 bp ssDNA

To optimize the CRISPR-Cpf1 genome-editing system in *S. cerevisiae*, we targeted for deletion of the *ADE2* gene, which has coloring properties to visually confirm the efficiency of genome editing. Three types of homologous DNA template donors for the CRISPR-Cpf1 editing system were designed, such as 1 kb double-strand DNA (dsDNA), 120 bp dsDNA, and 120 bp single-strand DNA (ssDNA). The results showed that the editing efficiency of the longer dsDNA template donor (1 kb) was significantly higher than that of the shorter (120 bp). Comparing the editing efficiency between different types of homologous DNA template donors, the ssDNA template donor was 12.5 times higher than the dsDNA template donor (Fig. 1). The results indicated that the ssDNA template donor for the CRISPR-Cpf1 system in *S. cerevisiae* could significantly improve the efficiency of genome editing.

Furthermore, we examined the electroporation parameters for genome editing in *S. cerevisiae*, including recovery period and plasmid concentration. The editing efficiency improved to 67.37 ± 2.34%, when using 500 ng plasmid amounts for electroporation and incubating for 48 h for cell recovery after electroporation (Table 1). These results indicated that using ssDNA as homologous template donors and recovering cells for longer periods had significant effects on genome-editing efficiency in the CRISPR-Cpf1 system in *S. cerevisiae*.

**Table 1:** Genome editing efficiency for the *ADE2* deletion

	Genome editing efficiency (%)
Recovery period (h)	
5	30.09 ± 2.04
24	47.76 ± 2.32**
48	55.02 ± 4.34**
Plasmid concentration (ng)	
250	55.02 ± 4.34
500	67.37 ± 2.34*

The genome-editing efficiency of the *ADE2* deleted strains were analyzed, including different recovery periods after electroporation and different concentration of plasmid for electroporation. For different recovery times, the concentration of plasmid DNA was 250 ng. For different plasmid concentrations, the recovery time was 48 h. The concentration of DNA template donors was 1 µg. The values shown represent at least three independent experiments (data are mean ± SD). The significant difference in 24 and 48 h recovery time from 5 h recovery time and in 500 ng plasmid transformed from 250 ng plasmid transformed was confirmed by Student’s t-test (*p < 0.05, **p < 0.01)

### Generation of *NTH1* and/or *HSP12* deletion strains by CRISPR-Cpf1

**Fig. 2:**
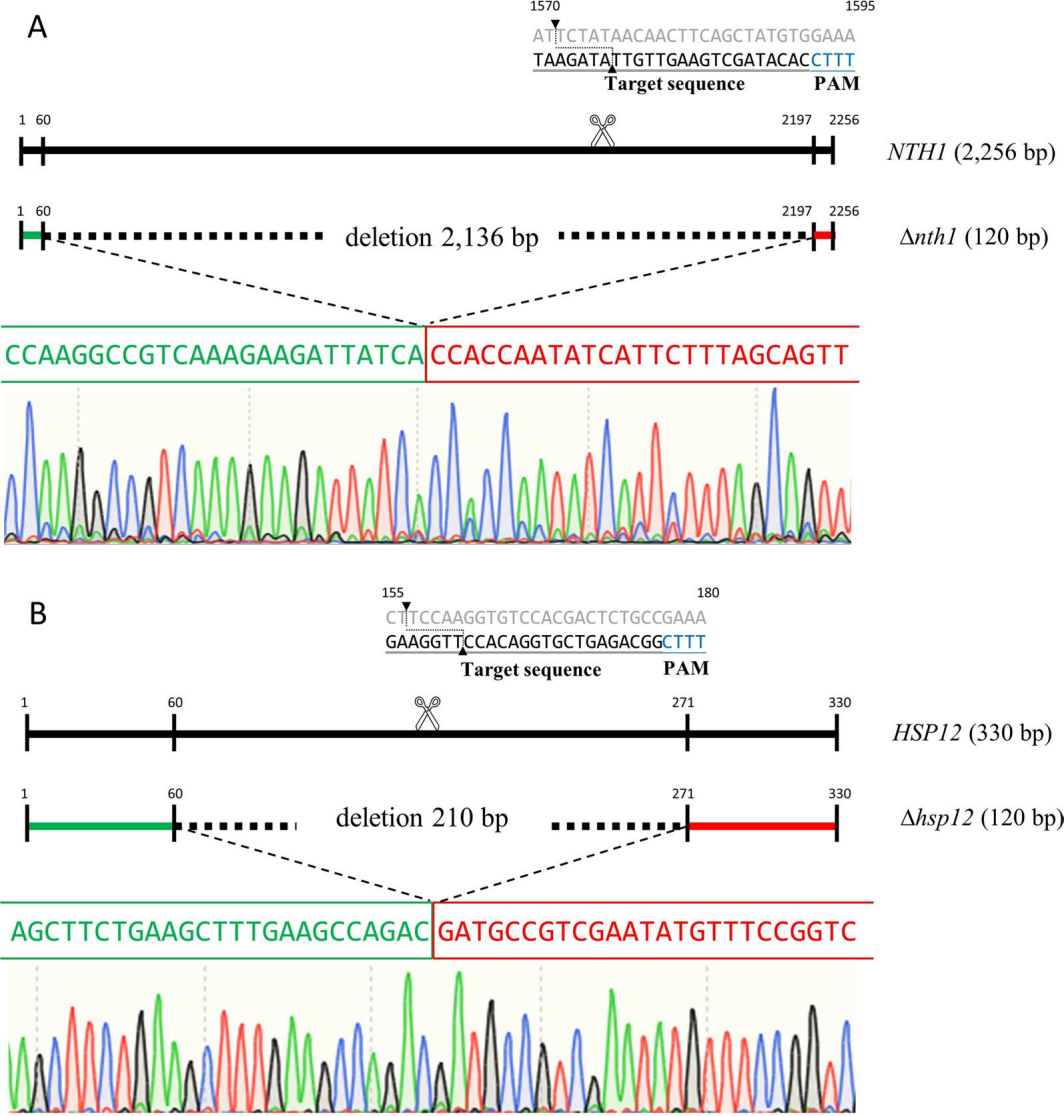
Gene targeting by CRISPR-Cpf1 for fragment deletions. The upper scheme showed the relative position of **A** *NTH1* and **B** *HSP12* using CRISPR-Cpf1 in *S. cerevisiae* system. The DNA sequencing spectrums shown below were for **A** the *NTH1* deletion strain and **B** the *HSP12* deletion strain

**Fig. 3:**
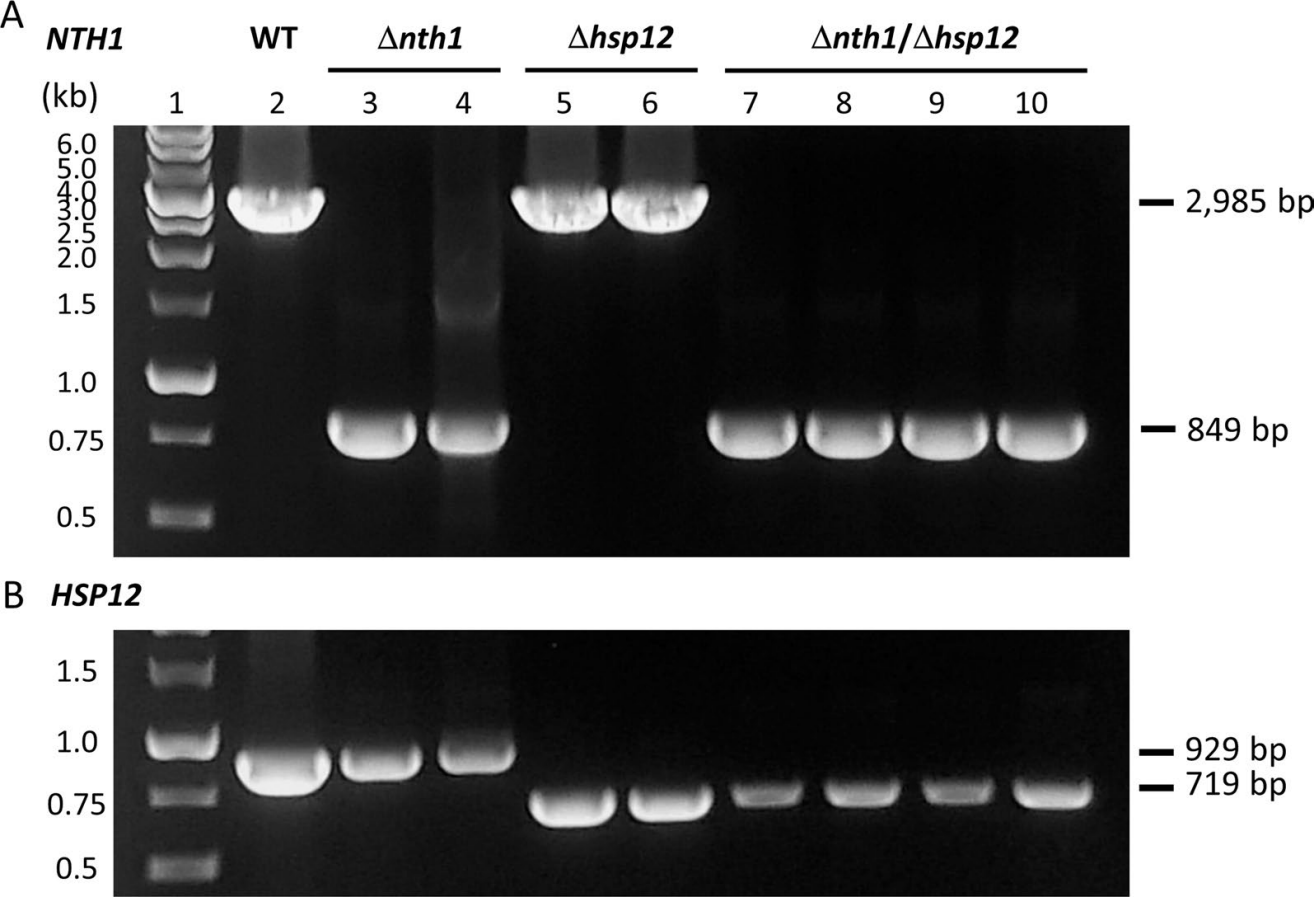
Agarose gel electrophoresis of colony PCR analysis for the genome-edited strains. PCR amplified the DNA contained **A** the *NTH1* gene by nth1CK1/nth1CK2 primer set and **B** the *HSP12* gene by hsp12CK1/ hsp12CK2 primer set. Lane 1, DNA ladders, lane 2, wild-type strain, lanes 3–4, *NTH1* deleted strain, lanes 5–6, *HSP12* deleted strain, lanes 7–10, *NTH1/HSP12* double deleted strain. PCR analysis was employed from two (Δ*nth1* and *Δhsp12*) or four (Δ*nth1*/Δ*hsp12*) separate colonies of edited yeast strains

Storage of frozen bread dough may lead to loss of cell viability of baker’s yeast and its baking capacity [1]. Trehalose can maintain the integrity of cell membranes and the native conformation of proteins in baker’s yeast under freezing stress [11]. Studies have demonstrated the correlation between the small heat-shock protein Hsp12p and yeast survival under freezing stress conditions [20]. To improve the baking capacity in baker’s yeast after long freezing storage in bread dough, the *NTH1* and/or *HSP12* genes of *S. cerevisiae* were deleted using the CRISPR-Cpf1 genome-editing system (Fig. 2). The *NTH1* deleted strain, the *HSP12* deleted strain, and the *NTH1*/*HSP12* double deleted strain were generated in this study. PCR analysis was used for the verification of *NTH1* and *HSP12* deletions (Fig. 3). As expected, PCR products in the deletion strains had smaller sizes than in WT strain. The trehalase activity of the edited strains was also analyzed. Compared with the WT strain, the trehalase activity of the *NTH1* deleted strains (Δ*nth1* and Δ*nth1/*Δ*hsp12*) were significantly reduced, but the trehalase activity of the *HSP12* deleted strain was not significantly altered (Table 2). Deletion of *NTH1* did not cause the complete abolishment of trehalase activity because other trehalase such as *NTH2* still presented in the *NTH1* deleted strains.

**Table 2:** Intracellular trehalose contents and trehalase activity of edited strains

#	Trehalose contents (mg/g CDW)	Trehalase activity (U/mg)
WT	45.4 ± 2.3	21.38 ± 1.92
Δ*nth1*	104.0 ± 2.3**	11.92 ± 1.22**
Δ*hsp12*	44.4 ± 2.5	22.31 ± 1.82
Δ*nth1*/Δ*hsp12*	112.9 ± 5.4**	13.63 ± 0.56**

The values shown represent at least three independent experiments (data are mean ± SD). The significant difference in the gene deleted strains from the wild-type strain was confirmed by Student’s t-test (**p < 0.01)

### Deletion *NTH1* increased intracellular trehalose contents

Trehalose is related to freezing-thawing stress tolerance in baker’s yeast [2]. To examine the intracellular trehalose contents in different knockout strains, cells were harvested in the stationary phase when trehalose synthesis is particularly intensive [31–33]. The results showed that the trehalose content of the *NTH1* deleted strain was 104 ± 2.3 mg/g cell dry weight (CDW), which was 2.3 times higher that of the wild-type strain 45.4 ± 2.3 mg/g CDW. However, the trehalose content was not increased in the *HSP12* deleted strain. Furthermore, the intracellular trehalose content of the *NTH1/HSP12* double knockout strain was 112.9 ± 5.4 mg/g CDW, which was 2.5 times higher than that of the wild-type strain (Table 2). The levels of trehalose were significantly higher in all *NTH1* deleted strains (Δ*nth1* and Δ*nth1/*Δ*hsp12*). It indicates that *NTH1*, but not *HSP12*, is important for the level of trehalose.

### *NTH1/HSP12* double deleted strains confer long-term freezing tolerance

**Fig. 4:**
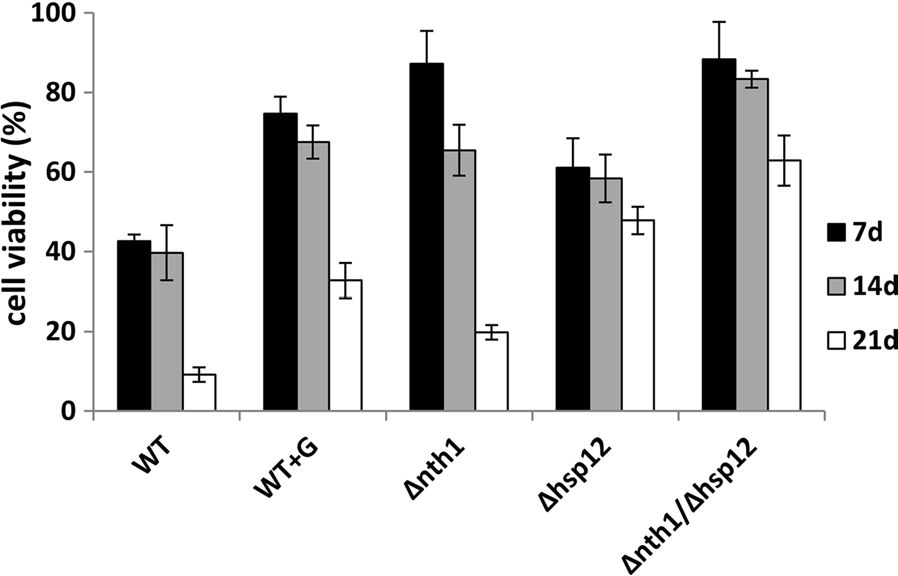
The cell viability of edited strains after freezing at − 20 °C. Data were derived from three separate experiments and are presented as the means ± SD. The symbol * indicated statistically significant differences of edited strains (**p < 0.01,*p < 0.05 using Student’s *t*-test) as compared to the wild-type strain

To investigate the freezing tolerance of the *NTH1* deleted and/or the *HSP12* deleted strains, the cell viability was analyzed after 7, 14, and 21 days of frozen storage. After 7 days of frozen storage, the cell viability of the wild-type strain was 42.6 ± 1.58% and 74.7 ± 4.26% without and with 30% glycerol as a cryoprotectant, respectively. However, it dropped to 9.15 ± 1.82% after 21 days of frozen storage without cryoprotectant. The cell viability of the *NTH1* deleted strain was 87.17 ± 8.32% and 65.46 ± 6.41% after 7 and 14 days of frozen storage, respectively; however, it dramatically decreased to 19.8 ± 1.85% after 21 days. It is suggested that the *NTH1* deletion strains could maintain cell viability for short-term frozen storage, but not for long-term frozen storage. The cell viability of the *HSP12* deleted strain was 61.05 ± 7.45%, 58.39 ± 6.02%, and 47.81 ± 3.46 after 7, 14, and 21 days of freezing, respectively. The *HSP12* deleted strains had lower viability than the *NTH1* deletion strains after 7 days of freezing, but higher viability after 21 days of freezing. Moreover, the cell viability of double genes deleted strains was 88.35 ± 9.41%, 83.33 ± 2.14%, and 62.87 ± 6.29% after 7, 14, and 21 days of freezing, respectively (Fig. 4). It is suggested that double deletion of *NTH1* and *HSP12* genes improved cell viability for long-term freezing. These data indicated that the trehalose content has a positive correlation with the viability of yeast cells for short-term freezing, and the absence of Hsp12p resulted in higher resistance to long-term freezing.

### *NTH1/HSP12* double deleted strain had enhanced leavening ability of frozen dough

**Fig. 5:**
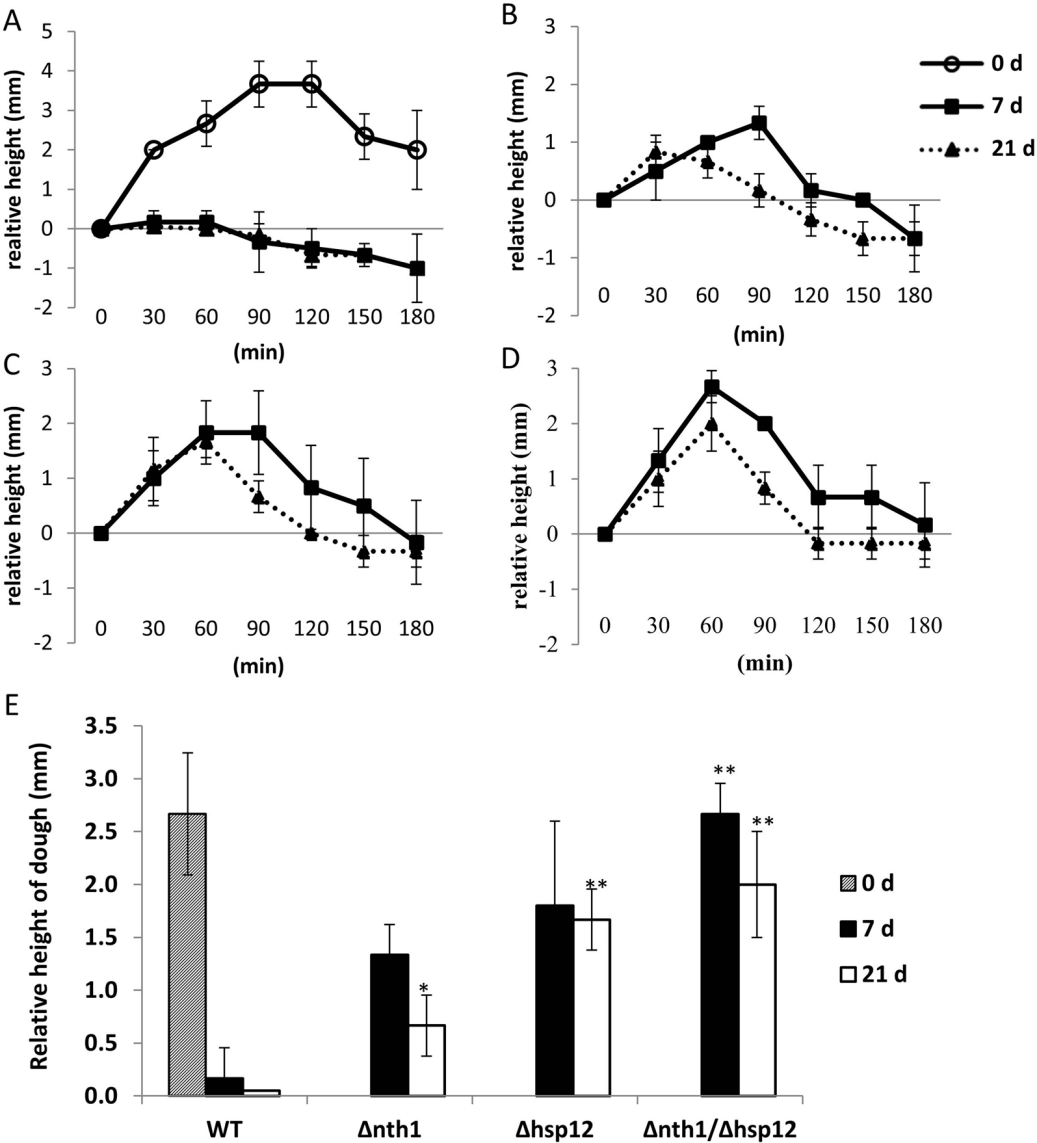
The leavening ability of the bread dough after 7 and 21 days of freezing. **A**–**D** Leavening profiles of bread dough used **A** the wild-type yeast strain, **B** the *NTH1* deleted yeast strain, **C** the *HSP12* deleted yeast strain, and **D** the *NTH1/HSP12* double deleted yeast strain. The height of the bread dough was measured after fermentation at 30 °C for 0–180 min. **E** The relative net height of the dough after fermentation for 60 min. Data was derived from three separate experiments and are presented as the means ± SD. The symbol * indicates statistically significant differences of edited strains (**p < 0.01,*p < 0.05 using Student’s *t*-test) as compared to the wild-type strain

An important fermentation characteristic of baker’s yeast used for frozen dough is leavening ability. We analyzed the leavening ability of bread dough containing different edited yeast strains after 7 and 21 days of freezing (Fig. 5). Our results showed that the bread dough lost the leavening ability after freezing and thawing compared to the fresh bread dough, suggesting that wild-type yeast died in frozen dough. The *NTH1* gene deletion alone and the *HSP12* deletion also slightly enhanced the leavening ability. Moreover, after 7 and 21 days of freezing, the bread dough with *NTH1/HSP12* double knockout strain significantly enhanced the leavening ability. These data suggested that *NTH1/HSP12* double deletion provides the best improvement of leavening ability upon freezing–thaw stress.

## Discussion

In this study, we established the high-efficiency CRISPR-Cpf1 genome-editing system in *S. cerevisiae*, and also constructed the edited strains with a double-gene deletion including the genes encoding the trehalose degradation enzyme and the membrane chaperone. The edited yeast increased the trehalose contents and the cell viability 21 days after freezing. The bread dough with edited yeast showed leavening ability after freezing, and it is indicated that precise editing of specific genes can improve freezing tolerance in yeast.

The quality of frozen dough depends on the ability of the yeast to generate carbon dioxide and the ability of the bread to retain carbon dioxide after fermentation. Decreased yeast viability is considered to be one of the main factors leading to the deterioration of dough quality [34]. Therefore, many studies have focused on generating yeast strains with improved growth or higher fermentation rates for frozen dough.

Maltose metabolism is related to the leavening ability in lean dough. Overexpression of maltose permease (encoded by *MAL62*) increases maltase activity and maltose metabolism, resulting in improved fermentation capacity of industrial baker’s yeast in lean dough [35]. Deletion of *NTH1* combined with *MAL62* overexpression enhances freezing tolerance and improves leavening ability after freezing [12]. Overexpression of *MAL62* and *TPS1* in an *NTH1*-deletion strain also enhances freezing tolerance and improves leavening ability [13]. The viability of yeast is also related to the content of several amino acids, such as glutamic acid, arginine, and proline [36]. Deletion of both *NTH1* and *PUT1* (a proline oxidase encoding gene) led to elevated levels of trehalose and proline, higher cell survival rate and higher dough-leavening ability after freezing [37]. The serine/threonine protein kinase (*SNF1* expressed) is an important regulator of yeast in response to stress. Overexpression of *SNF1* is effective in enhancing the cell tolerance and fermentation capacity of baker’s yeast in freezing, which may be related to the upregulated proteasome, altered metabolism of carbon sources and protectant molecules, and changed cell membrane components [38, 39]. Ycp4p is predicted to be palmitoylated, a posttranslational modification typical of membrane-binding proteins involved in signal transduction. Disruption of *YCP4* enhances freeze–thaw tolerance in baker’s yeast [40]. In this study, the yeast strains were manipulated with *NTH1* and/or *HSP12* deletion by genome-editing technology, and the leavening ability was increased in the bread dough with *NTH1/HSP12* double deletion strain after freezing (Fig. 5).

Chaperone proteins are essential for maintaining the functionality of the cellular proteome to prevent the aggregation of unfolded protein under stress conditions, such as heat, low oxygen, heavy metals, UV radiation, freezing and thawing. The *HSP12* deletion mutant is more resistant to freezing, and overexpression of Hsp12p in the *TPS1* deletion strain increases the resistance to freezing stress [20]. It is contradictory. Both deletion and overexpression of *HSP12* gene improve resistance to freezing. Hsp12p is monomeric and unfolded in solution, and it is most likely a membrane chaperone expressed during stresses. Hsp12p gains in structure in the presence of specific lipids (PiP_2_) leading to membrane rigidification [41]. Membrane fluidity is higher, yeast cells survive better under freeze–thaw stress [42]. The freeze/thaw process results in a rigidifying effect on the cell membrane and cell adaptability to freeze/thaw-induced stress could be dependent on their initial membrane fluidity. Hsp104p contributes to freeze–thaw tolerance by maintaining ubiquitin-proteasome system activity via the disaggregation of aggregated proteins. Disruption of *HSP104* caused a reduction in cell viability [43].

The *NTH1* deletion strain with higher trehalose levels (Table 2) could maintain cell viability for short-term frozen storage, but not for long-term frozen storage (Fig. 4). The *HSP12* deleted strain, without increased trehalose levels (Table 2), was less viable than the *NTH1* deletion strain for short-term frozen storage, but more viable for long-term frozen storage (Fig. 4). Disruption of *NTH1* contributed to an accumulation of trehalose and disruption of *HSP12* contributed to long-term survival upon freezing stress. In this study, we demonstrated that the *NTH1/HSP12* double deleted strain, with high levels of trehalose content and the long-term viability under frozen conditions, enhanced the leavening ability of frozen dough (Fig. 5d). However, compared to fresh dough with the wild-type strain (Fig. 5a), the leavening ability and fermentation time still needed to be improved (Fig. 5d).

In this study, we employed the CRISPR-Cpf1 system to generate mutant strains with precise gene deletion. We manipulated the *NTH1* and *HSP12* gene double deletions by simultaneously expressing Cpf1 and crRNAs based editing on two loci. It was successfully achieved and it was efficient. CRISPR has been hailed as a promising technology because it can accurately insert and alter DNA with targeted specificity and relatively easy implementation. However, recent advances in genome-editing technologies have led to a new era, and the technologies involve precise gene editing without the transfer of foreign genes. The genome-editing technology can be applied to improve strain characteristics and it will enhance the application and development of yeast in the food industry.

## Conclusions

In summary, we constructed an efficient CRISPR-Cpf1 genome-editing system, including two direct repeats flanking the crRNA sequence, 120 bp single-strand homologous templates, 48 h recovery period after electroporation, and higher transformed plasmids concentration. *NTH1*or/and *HSP12* knockout in *S. cerevisiae* by the optimized CRISPR-Cpf1 genome-editing system enhanced the freezing tolerance and improved the leavening ability after freezing and thawing stress. Deleting *NTH1* would introduce the increase of the trehalose contents and achieve a protective role in shorter-term freezing storage. Deleting *HSP12* in the combination with deleting *NTH1* would strengthen the freezing tolerance and protect the cell viability from higher levels of death in longer-term freezing. It provides valuable insights for breeding novel *S. cerevisiae* strains for the baking industry through a more precise, speedy, and economic genome-editing system.

## Methods and materials

### Strains and plasmids

The *S. cerevisiae* wild-type strain BCRC 21447 used in this study was obtained from Bioresource Collection and Research Center. The plasmid vectors pUDC175 and pUDE710 were generated by Dr. Jean-Marc Daran (Addgene plasmid #103019 and #103020) [44]. The genetic properties of *S. cerevisiae* strains and plasmids used in the present study are summarized in Table 3.

**Table 3:** Characteristics of strains and plasmids used in this study

Strains or plasmids	Characteristics	Reference or source
*S. cerevisiae* BCRC21447	*MATa/a* industrial brewer’s top yeast	Bioresource collection and research center
*S. cerevisiae* Δ*nth1*	*MATa/a*, Δ*nth1 +* pBCoN1	This study
*S. cerevisiae* Δ*hsp12*	*MATa/a*, Δ*hsp12 +* pBCoH1	This study
*S. cerevisiae* Δ*nth1*/Δ*hsp12*	*MATa/a*, Δ*nth1*Δ*hsp12 +* pBCoNH1	This study
pUDC175	*E. coli/S. cerevisiae* shuttle vector, containing Amp^+^, TRP1^+^, *Fn*Cpf1	Świat et al. [23]
pUDE710	*E. coli/S. cerevisiae* shuttle vector, containing Amp^+^, KanMX^+^, crADE2-crHIS4	Świat et al. [23]
pBCo1	*E. coli/S. cerevisiae* shuttle vector, containing Amp^+^, KanMX^+^, *Fn*Cpf1, crADE2	This study
pBCoN1	*E. coli/S. cerevisiae* shuttle vector, containing Amp^+^, KanMX^+^, *Fn*Cpf1, crNTH1.2	This study
pBCoH1	*E. coli/S. cerevisiae* shuttle vector, containing Amp^+^, KanMX^+^, *Fn*Cpf1, crHsp12.1	This study
pBCoNH1	*E. coli/S. cerevisiae* shuttle vector, containing Amp^+^, KanMX^+^, *Fn*Cpf1, crNTH1.2-crHsp12.1	This study

### Media and growth conditions

*Escherichia coli* Top10 strain, used for DNA amplification, was grown at 37 °C in a LB medium (10 g/L tryptone, 5 g/L yeast extract, and 10 g/L NaCl). *S. cerevisiae* was grown at 30 °C in a YPD medium (1% yeast extract, 2% peptone, and 2% glucose). When the selection was required, antibiotics were added with 100 mg/L ampicillin for *E. coli*, and 200 mg/L G418 for *S. cerevisiae.*

### Plasmid construction

Yeast genomic DNA was separated from *S. cerevisiae* BCRC 21447 using a DNA microprep kit (D4301, Zymo Research Corp.) Primers used in this study were purchased from Genomics BioSci &Tech. Co., Ltd. The plasmid pBCo1was applied to express *Fn*Cpf1cassette and contained crRNA sequence crADE2 targeting gene for *ADE2* gene deletion. The plasmid pBCoN1 was applied to express *Fn*Cpf1cassette and contained crRNA sequence crNTH1.2 targeting gene for *NTH1* gene deletion. The plasmid pBCoH1 was applied to express *Fn*Cpf1 cassette and contained crRNA sequence crHSP12.1 targeting gene for *HSP12* gene deletion. The plasmid pBCoNH1 was applied to express *Fn*Cpf1cassette and contained crRNA sequences crNTH1.2 and crHSP12.1 targeting gene for *NTH1* and *HSP12* gene deletion, respectively. The crRNA sequences were designed by CHOPCHOP website (http://chopchop.cbu.uib.no/) and based on the following principles: (1) the PAM sequence of *Fn*Cpf1 for crRNA is 5′-TTTV-3′ (V = A/G/C), (2) the length of direct repeats (DR) is 19 nt, and (3) crRNA is flanked by two DRs at 5’ and 3′ end [23].

### Transformation and genome editing in yeast

All yeast transformations were performed using the lithium acetate protocol as previously described [44]. The synthesized homologous DNA templates contained 60 bp of DNA upstream and downstream of the target gene (Table 4). Oligonucleotides were purchased from Integrated DNA Technologies (IDT) Int. The edited strains with deleted *ADE2* were visible colored colonies since the *ADE2* gene was essential for adenine biosynthesis, and its deletion resulted in adenine auxotrophy and red colonies [45]. The efficiency of genome editing was calculated by the percentage of red colonies to total colony numbers. The genome editing of *NTH1* and *HSP12* was confirmed by colony PCR analysis with nth1CK1/nth1CK2 and hsp12CK1/hsp12CK2 primer sets and DNA sequencing.

**Table 4:** Target genes, primers, and homologous templates used in this study

Gene	Targeted sequence	PAM	Primer sequence for colony PCR	ssDNA template sequence
*ADE2*	CCGGTTGTGGTATATTTGGTGTGGA	TTTC	F: TCTAAGTACATCCTACTATAACAATC R: GGACACTTATATGTCGAGCAAGA	TTACTTGTTTTCTAGATAAGCTTCGTAACCGACAGTTTCTAACTTTTGTGCTTTGACAAGACAATCATACGTCCCAATTGTCCCCCTCCTAATATACCAACTGTTCTAGAATCCAT
*NTH1*	CACATAGCTGAAGTTGTTATAGAAT	TTTC	F: GACAGGAGCATCGTGTAGGA R: CTACCTAGGAGCGGCCAA	CTATAGTCCATAGAGGTTTCTTTCTTGAGGCCTTAAACTGCTAAAGAATGATATTGGTGGTGATAATCTTCTTTGACGGCCTTGGGCTACCGGTCCTTGGCTTGTATTAACTTGACTCAT
*HSP12*	GGCAGAGTCGTGGACACCTTGGAAG	TTTC	F: CCCACAAACACAGACCAGAA R: CGCGGAAATTGATGACGAA	ATGTCTGACGCAGGTAGAAAAGGATTCGGTGAAAAAGCTTCTGAAGCTTTGAAGCCAGACGATGCCGTCGAATATGTTTCCGGTCGTGTCCACGGTGAAGAAGACCCAACCAAGAAGTAA

### Trehalase activity assay

Trehalase activity was measured by monitoring the production of glucose from trehalose. Yeast cells were harvested by centrifugation and washed twice with distilled water. The cells were suspended in extraction buffer and disrupted with glass beads. The supernatant was used as a crude extract after removing debris by centrifugation at 12,000×*g* for 10 min at 4 °C. Trehalase activity in supernatants was measured using the trehalase assay kit (ARG82017, Arigo Biolaboratories Corp.). One unit of trehalase activity was the enzyme that generated 1 µmol of glucose per minute.

### Assay of the intracellular trehalose content

Intracellular trehalose of yeast cells was extracted as briefly described as follows [12]. Yeast cells were harvested by centrifugation and washed twice with distilled water. Trehalose was extracted from 0.1 g cell pellets with 4 mL of 5% (w/v) cold trichloroacetic acid for 45 min with shaking. Extraction was repeated once more, and supernatants from the two extractions were combined and used for measuring the trehalose content by the trehalose assay kit (K-TREH, Megazyme ltd.). The CDW was determined by drying fresh yeast cells overnight at 85 °C.

### Determination of cell viability

For the freeze–thaw stress, yeast cells were cultured in a YPD medium at 30 °C and harvested in the stationary phase. After microscopic counting of viable cells by trypan blue staining using a hemocytometer, cells were aliquoted into cryotubes and frozen at − 20 °C for various periods. The number of cells was counted after thawing the frozen cell samples at 30 °C for 30 min. The survival ratio was calculated from the number of living cells before and after freezing.

### Determination of leavening ability

The composite of bread dough was 10 g of wheat flour, 5 mL of water, 1.25 g of sugar, 0.25 g of salt, and 0.1 mL of fresh yeast cells at 0.3 g/mL. All the ingredients were mixed properly, and each dough was put into a 50 mL graduated cylinder. The height of the dough was recorded every 30 min up to 3 h. One group of dough was prepared with fresh yeast samples as a positive control, while another group of dough was prepared without any yeast samples as a negative control. The experimental groups of dough with edited yeast cells were stored at − 20 °C for 7 or 21 days. The frozen dough was thawed at 30 °C for 30 min and then tested for the leavening ability. The height of the dough was measured from the graduated surface of the graduated cylinder before and after fermentation, and the net increased height was calculated [46].

### Statistical analysis

All the experiments were performed individually at least three times, and the data reported were mean values ± SD. The differences between the edited strains and the parental strain were confirmed by Student’s t-test. Differences at *p* < 0.05 were considered statistically significant.

## Data Availability

Not applicable.

## Abbreviations

ADE2: Phosphoribosylaminoimidazole carboxylase 2
CDW: Cell dry weight
CRISPR: Clustered regularly interspaced short palindromic repeats
crRNA: crispr RNA
DR: Direct repeats
*Fn*Cpf1: Cpf1 variants from *Francisella novicida*
HSP12: Heat shock protein 12
NTH1: Neutral trehalase 1
PAM: Protospacer adjacent motif
SD: Standard deviation
